# Modelling of surface reactions and diffusion mediated by bulk diffusion

**DOI:** 10.1098/rsta.2022.0367

**Published:** 2023-12-25

**Authors:** Fernando P. Duda, Francisco S. Forte Neto, Eliot Fried

**Affiliations:** ^1^ Programa de Engenharia Mecânica, COPPE, Universidade Federal do Rio de Janeiro, Cidade Universitária, Rio de Janeiro, CEP 21941-972, RJ, Brazil; ^2^ Mechanics and Materials Unit, Okinawa Institute of Science and Technology Graduate University, Onna, Okinawa 904-0495, Japan

**Keywords:** bulk-surface partial-differential equations, internal constraints, diffusion, reaction kinetics, stability, pattern formation

## Abstract

We develop a continuum framework applicable to solid-state hydrogen storage, cell biology and other scenarios where the diffusion of a single constituent within a bulk region is coupled via adsorption/desorption to reactions and diffusion on the boundary of the region. We formulate content balances for all relevant constituents and develop thermodynamically consistent constitutive equations. The latter encompass two classes of kinetics for adsorption/desorption and chemical reactions—fast and Marcelin–De Donder, and the second class includes mass action kinetics as a special case. We apply the framework to derive a system consisting of the standard diffusion equation in bulk and FitzHugh–Nagumo type surface reaction–diffusion system of equations on the boundary. We also study the linear stability of a homogeneous steady state in a spherical region and establish sufficient conditions for the occurrence of instabilities driven by surface diffusion. These findings are verified through numerical simulations which reveal that instabilities driven by diffusion lead to the emergence of steady-state spatial patterns from random initial conditions and that bulk diffusion can suppress spatial patterns, in which case temporal oscillations can ensue. We include an extension of our framework that accounts for mechanochemical coupling when the bulk region is occupied by a deformable solid.

This article is part of the theme issue ‘Foundational issues, analysis and geometry in continuum mechanics’.

## 1. Introduction

The interaction between bulk diffusion and surface processes, including chemical reactions and diffusion at the boundary of the bulk region, is crucial in many scientific and technological fields. One instance is the development of solid-state hydrogen storage materials based on metal hydrides, where the adversely slow kinetics of hydrogen uptake and release by metals are attributed to this interplay. The kinetics of hydrogen uptake from a gaseous environment involves the adsorption and dissociation of hydrogen molecules and hydrogen penetration into and diffusion within the solid, as well as a metal-hydride phase transition from a low-content hydrogen phase to a higher-content hydrogen phase (see, for instance, Martin *et al.* [1]). Another complex phenomenon that relies on the interplay of bulk diffusion and surface processes is protein pattern formation in cell biology (see, for instance Burkart *et al.* [2]). In this case, the dynamics of intracellular signalling molecules, such as Cdc42, which exist in inactive and active forms (see, for instance, Gomez *et al.* [3]), capture essential features of the phenomenon. The inactive form diffuses through the cytoplasm and membrane, undergoing attachment, detachment and interconversion with the active form, which can also diffuse.

Extensive research has been conducted on reaction–diffusion systems that describe the interplay between diffusion in a bulk region and chemical reactions on the boundary of the region. Typically, these systems are described by partial-differential equations in bulk that are coupled with surface ordinary- or partial-differential equations on the boundary depending on whether surface diffusion is considered. However, the current literature mainly focuses on analysing, computing and applying these coupled equations rather than deriving them. Therefore, it often needs to be clarified how the chemical processes are interconnected and how to generalize them to include other physical processes such as mechanical deformation and flow. Burkart *et al.* [2] have emphasized the need for a unified theoretical approach to multi-physics problems in the context of pattern formation in cells. Continuum theories for multi-physics problems involving, among other things, bulk diffusion, mechanical deformation and flow have been developed and applied with considerable success. However, the consideration of surface reaction and diffusion has received less attention. To bridge this gap, we propose a continuum framework for describing bulk diffusion coupled with surface chemical reactions and diffusion.

We focus on a material system composed of a single bulk constituent present in a region R and multiple surface constituents at the boundary of ∂R. The system undergoes bulk diffusion within R and surface diffusion and chemical reactions on ∂R, and these processes are coupled by the consumption/generation of the bulk constituent through adsorption/desorption. We describe all surface chemical processes using sorption and reaction mechanisms consisting of elementary steps, where the participation of the bulk constituent is limited to the elementary steps of the sorption mechanism. Meanwhile, the adsorbed form of the bulk constituent can participate in all the elementary steps constituting the sorption and reaction mechanisms. With these considerations in mind, we (i) develop a theoretical framework for describing the interplay between bulk diffusion, adsorption and desorption, and surface diffusion and chemical reactions; (ii) formulate boundary conditions for the bulk diffusion problem; and (iii) explore an example in which bulk diffusion is mediated by a FitzHugh–Nagumo type surface reaction–diffusion process.

To achieve our objectives, we introduce constituent content balances and a mechanical version of the second law of thermodynamics, namely, the free-energy imbalance. The concepts of free energy, chemical power and constituent chemical potential are introduced through the free-energy imbalance. Subsequently, we introduce a constitutive theory consistent with thermodynamics. The constitutive theory hinges on assuming that (i) the production rate of a constituent is the sum of contributions from each elementary step in which it participates; and (ii) the production rate of a constituent in an elementary reaction is determined by the rate of the elementary reaction multiplied by its stoichiometric coefficient in that reaction. We are thus led to the notion of affinity of an elementary chemical reaction and the consideration of two types of kinetics for an elementary reaction: fast or Marcelin–De Donder. In the former case, the affinity of a reaction is constrained to be null, with the reaction rate being a Lagrange multiplier necessary to maintain that constraint. In the latter case, the reaction rate of an elementary reaction depends, in contrast, on its affinity. Under suitable constitutive assumptions, the Marcelin–De Donder kinetics reduces to the classical mass action kinetics. Our developments allow for the possibility that a surface constituent may be constrained to occupy its lattice, resulting in the splitting of its chemical potential into two parts. One of these parts is constitutively determined, while the other is not and can be seen as a Lagrange multiplier associated with the lattice constraint.

Our discussion of the boundary condition for bulk diffusion centres on the Langmuir adsorption/desorption mechanism, a single-step process involving the bulk constituent and its adsorbed form. Specifically, we derive a Dirichlet-type boundary condition that specifies the value of the diffusing substance at the boundary, provided that the corresponding elementary reaction is so fast that its affinity can be constrained to vanish. If, however, the kinetics of the elementary reaction do not meet this assumption, we instead derive a Robin-type boundary condition whereby the flux of the diffusing constituent is prescribed by the Marcelin–De Donder kinetics.

We then derive a system of equations describing bulk diffusion coupled with a surface reaction–diffusion process of the FitzHugh–Nagumo type. To achieve this, we incorporate the chemical reaction mechanism suggested by Malevanets & Kapral [4]. In addition, we assume that the adsorption/desorption process occurs very rapidly, leading to a Dirichlet-type boundary condition for bulk diffusion. We then perform a linear stability analysis around a homogeneous steady state in a spherical region, and establish sufficient conditions for the occurrence of instability driven by surface diffusion. Our findings are verified through numerical simulations of the nonlinear equations, from which we infer that surface diffusion-driven instability leads to the emergence of steady-state spatial patterns from random initial conditions. We also present the spherically averaged structure factor for the steady-state patterns obtained for representative choices of parameters.

Our approach can be extended to incorporate other physically salient effects. We illustrate this for situations in which the bulk region is occupied by a deformable solid through which a single mobile constituent can diffuse, accounting for chemical reactions and diffusion on the boundary of the solid.

## 2. Preliminaries

Given an open region R in three-dimensional Euclidean point space with closed boundary ∂R, we suppose that R∪∂R is occupied by a material system composed of N+1 chemical constituents: a single bulk chemical constituent B and N surface chemical constituents $A_{I},\;I=1,\ldots ,N$. However, the bulk constituent can diffuse within R and be adsorbed by or desorbed from ∂R, and the surface constituents can undergo surface diffusion and participate in a process comprising M elementary chemical reactions taking place on ∂R. We also allow for the presence of an environmental constituent E, which can be adsorbed by or desorbed from ∂R. The surface constituents include the adsorbed forms of both the bulk and environmental constituents. The composition of the system under consideration is determined by the number density $n_{B}$ of the bulk constituent, gauged per unit volume, and the number density $n_{I}$ of constituent $A_{I},\;I=1,\ldots ,N$, gauged per unit area.

We adopt the standard notation of continuum mechanics throughout this article. Specifically, ∇,  div and △ represent the gradient, divergence and Laplacian operators, and $\dot{\varphi }$ denotes the time derivative of a field φ. In addition, we rely on three additional operators, namely, $\nabla_{\;S}\;$, ${\text{ div}}_{\;S\;}$ and ${△}_{S}$, which, respectively, indicate the surface gradient, surface divergence and Laplace–Beltrami operators. Detailed definitions of these operators can be found in other sources, such as Fried & Gurtin [5].

## 3. Basic laws

The basic principles governing the evolution of this system are comprised by the content balances for each constituent and a free-energy imbalance. These laws can be expressed in integral forms for the bulk and surface parts, P and A, of the region R and its boundary ∂R, respectively. P is an open region interior to R, and A can be envisioned as an open pillbox-type region containing a subsurface of ∂R and adjacent portions of both R and the surrounding region exterior to R. For brevity, we present the strong forms of the basic laws that underpin our framework. Full statements of the basic laws and the details of their localization are provided as supplementary material. We henceforth denote by m the outward unit normal to a part P and by n the outward unit normal to ∂R.

### (a) Content balance for the bulk constituent

The strong form of the content balance for the bulk constituent B yields the equation

3.1 $${\dot{n}}_{B}=-\text{ div}\;{ȷ}_{B}+m_{B}\;\text{on}\;R,$$

where ${ȷ}_{B}$ and $m_{B}$ denote the flux and supply of constituent B, respectively, and the equation

3.2 $${ȷ}_{B}\cdot n+r_{B}=0\;\text{on}\;\partial R,$$

where ${ȷ}_{B}\cdot n$ and $r_{B}$ represent the outflow from R and rate of production on ∂R, respectively, of constituent B. Environmental supplies and inflows of constituent B have been neglected leading to (3.1) and (3.2).

### (b) Content balances for the surface and environmental constituents

While the strong form of the content balance for surface constituent $A_{I}$, I=1,…,N, yields the equation

3.3 $${\dot{n}}_{I}=-{\text{ div}}_{\;S\;}{ȷ}_{I}+r_{I}+m_{I}\;\text{on}\;\partial R,$$

where ${ȷ}_{I}$, $r_{I}$ and $m_{I}$ denote, respectively, the surface flow, the rate of production and rate of supply of constituent $A_{I}$, the strong form of the content balance for the environmental constituent yields the equation

3.4 $$-{ȷ}_{E}\cdot n+r_{E}=0\;\text{on}\;\partial R,$$

where ${ȷ}_{E}\cdot n$ and $r_{E}$ represent the outflow from R and rate of production on ∂R, respectively, of constituent E.

### (c) Summary of the local equations

For easy reference, we summarize the equations arising from the balances for the bulk and surface constituents:

3.5 $$\begin{array}{l} \;\;\;{\dot{n}}_{B}=-\text{ div}\;{ȷ}_{B}+m_{B}\;\;\;\text{on}\;R;\\ \begin{array}{l} {ȷ}_{B}\cdot n+r_{B}=0,\\ {\dot{n}}_{I}=-{\text{ div}}_{\;S\;}{ȷ}_{I}+r_{I}+m_{I},\;I=1,\ldots ,N,\\ {ȷ}_{E}\cdot n-r_{E}=0, \end{array}\}\;\text{on}\;\partial R. \end{array}\}$$

### (d) Free-energy imbalance

The free-energy imbalance serves as a mechanical version of the second law of thermodynamics and applies to both bulk and surface parts in the present context. The strong form of this law yields the inequality

3.6 $$\dot{\psi }-\mu_{B}{\dot{n}}_{B}+{ȷ}_{B}\cdot \zeta_{B}\leq 0\;\text{in}\;R,$$

where ψ is the free-energy density and $\mu_{B}$ is the chemical potential of constituent B, both gauged per unit volume, and

3.7 $$\zeta_{B}=\nabla \mu_{B}$$

denotes the gradient of the chemical potential of constituent B, and the inequality

3.8 $${\dot{\psi }}_{S}-\sum_{I=1}^{N}(\mu_{I}{\dot{n}}_{I}-{ȷ}_{I}\cdot \zeta_{I}-\mu_{I}r_{I})+\mu_{B}r_{B}+\mu_{E}r_{E}\leq 0\;\text{on}\;\partial R,$$

where $\psi_{S}$ is the surface free-energy density, gauged per unit area, $\mu_{I}$ is the chemical potential of constituent I, I=1,…,N, and

3.9 $$\zeta_{I}=\nabla_{\;S}\;\mu_{I}$$

denotes the surface gradient of the chemical potential of the constituent $A_{I},\;I=1,\ldots ,N$.

## 4. Bulk constitutive theory

Guided by (3.6), we identify $n_{B}$ and ${ȷ}_{B}$ as independent constitutive variables and ψ, $\mu_{B}$ and $\zeta_{B}$ as dependent constitutive variables. With these provisions, we find that ψ must be independent of ${ȷ}_{B}$ and thus given by a relation of the form

4.1 $$\psi =\tilde{\psi }(n_{B})$$

and $\mu_{B}$ must be generated from derivative of the response function $\tilde{\psi }$ through the relation

4.2 $$\mu_{B}=\frac{\partial \tilde{\psi }(n_{B})}{\partial n_{B}},$$

and, thus, must also be independent of ${ȷ}_{B}$; the response function ${\hat{\zeta }}_{B}$ must satisfy the residual free-energy inequality

4.3 $${\hat{\zeta }}_{B}(n_{B},{ȷ}_{B})\cdot {ȷ}_{B}\leq 0.$$

## 5. Surface constitutive theory

We begin by introducing the following assumptions pertaining to the origin of the source terms $r_{B}$, $r_{I},\;I=1,\ldots ,N$, and $r_{E}$ in the pointwise balances (3.2)–(3.4) on ∂R:

— The source terms $r_{B}$, $r_{I}$, I=1,…,N and $r_{E}$ result from M elementary reactions (see, for instance, Svehla [6])
  5.1 $$\sum_{I=0}^{N}\alpha_{I}^{j}A_{I}⇌\sum_{I=0}^{N+1}\beta_{I}^{j}A_{I},\;j=1,\ldots ,M,$$
  that take place on ∂R and encompass adsorption or desorption processes involving the bulk and environmental constituents, as labelled by $B=:A_{0}$ and $E=:A_{N+1}$. Here and henceforth, $\alpha_{I}^{j}\geq 0$ and $\beta_{I}^{j}\geq 0$ denote the forward and backward stoichiometric coefficients of the chemical constituent $A_{I}$ in the jth reaction, j=1,…,M.
— The net production rate $r_{I}$ of constituent $A_{I}$, with I=0,…,N+1, is given by
  5.2 $$r_{I}=\sum_{j=1}^{M}r_{I}^{j},\;\text{with}\;r_{I}^{j}=(\beta_{I}^{j}-\alpha_{I}^{j})r^{j},$$
  where $r_{I}^{j}$ is the net production rate of constituent $A_{I}$ in the jth reaction and $r^{j}$ is the rate of the jth reaction.

By (5.2), the net production rate of a constituent is obtained by adding the contributions from each elementary reaction, which in turn is given by the net variation of the number of molecules of that constituent multiplied by the rate of that reaction (see, for instance, Feinberg [7]). More importantly, the pointwise consequence (3.8) of free-energy imbalance on ∂R can be recast as follows:

5.3 $${\dot{\psi }}_{S}-\sum_{I=1}^{N}(\mu_{I}{\dot{n}}_{I}-{ȷ}_{I}\cdot \zeta_{I})-\sum_{j=1}^{M}f_{j}r^{j}\leq 0,$$

where the affinity $f_{j}$ of the jth reaction, j=1,…,M, is given by

5.4 $$f_{j}=\sum_{I=0}^{N+1}(\alpha_{I}^{j}-\beta_{I}^{j})\mu_{I}.$$

We stipulate that the bulk constituent $B=A_{0}$ participates in (5.1) solely through the reaction $R_{1}$, which in turn we take to be of the form

5.5 $$A_{0}+A_{1}⇌A_{2},$$

where $A_{1}$ represents a vacant surface site, itself treated as a chemical constituent, and $A_{2}$ represents the adsorbed constituent corresponding to B. By (5.5), which corresponds to the Langmuir adsorption/desorption mechanism (see, for instance, Houston [8]), the only non-vanishing stoichiometric coefficients of the sorption reaction $R_{1}$ are $\alpha_{0}^{1}=\alpha_{1}^{1}=1$ and $\beta_{2}^{1}=1$. Also, the bulk constituent B participates only in the sorption reaction, with the consequence that $\alpha_{0}^{j}=\beta_{0}^{j}=0$ for the remaining reactions (j≠1). Thus, (5.2) implies that

5.6 $$r_{B}=-r^{1}.$$

Reasoning analogously, we stipulate that the environmental constituent $E=A_{N+1}$ participates in (5.1) solely through the reaction $R_{M}$, which in turn we take to be of the form

5.7 $$A_{N+1}+A_{N}⇌A_{N-1},$$

where $A_{N}$ represents a vacant surface site, itself treated as a chemical constituent, and $A_{N-1}$ represents the adsorbed constituent corresponding to E. By (5.7), which also corresponds to the Langmuir adsorption/desorption mechanism, the only non-vanishing stoichiometric coefficients of the sorption reaction $R_{M}$ are $\alpha_{N+1}^{M}=\alpha_{N}^{M}=1$ and $\beta_{N-1}^{M}=1$ and that $\alpha_{N+1}^{j}=\beta_{N+1}^{j}=0$ for the remaining reactions (j≠M), which by (5.2) imply that

5.8 $$r_{E}=-r^{M}.$$

We next use (5.3) as a guide for identifying lists of independent and dependent constitutive variables. Specifically, we consider the independent constitutive variables to be given by the list

5.9 $$(n_{1},\ldots ,n_{N},{ȷ}_{1},\ldots ,{ȷ}_{N},r^{1},\ldots ,r^{M}).$$

If the variables in the foregoing list were unconstrained, the list of dependent constitutive variables would be given by $\left(\psi_{S},\mu_{1},\ldots ,\mu_{N},\zeta_{1},\ldots ,\zeta_{N},f_{1},\ldots ,f_{M}\right)$. Here, however, we allow for the presence of internal constraints.

If $f_{j}=0$, j∈{1,…,M}, then $r^{j}$ is not included in the list (5.9) of independent variables. This condition amounts to stipulating that the jth reaction occurs very rapidly relative to all other reactions, and, hence, that local chemical equilibrium is attained instantaneously.

### (a) Internal constraints

We will now present a treatment applicable to situations in which certain chemical constituents are restricted to occupying corresponding lattice sites. This treatment relies on the notion of internal constraints to address the lattice constraint. For simplicity, we consider a scenario wherein only the chemical constituent participating in the sorption reactions (5.5) and (5.7) are constrained.

Following Gurtin *et al.* [9, §72], the adsorbed constituent $A_{2}$ and $A_{N-1}$ are constrained to occupy lattice sites with respective number densities $\bar{n}>0$ and $\bar{\bar{n}}>0$, whereby lattice constraints

5.10 $$n_{1}+n_{2}=\bar{n}\;\text{and}\;n_{N-1}+n_{N}=\bar{\bar{n}},$$

hold, and to diffuse substitutionally, so that the substitutional flux constraints

5.11 $${ȷ}_{1}+{ȷ}_{2}=0\;\text{and}\;{ȷ}_{N-1}+{ȷ}_{N}=0$$

hold. It accordingly follows from (3.3) and (5.10)${\;}_{1}$ that $r_{1}+m_{1}+r_{2}+m_{2}=0$, whereby it seems natural to enforce the supplemental condition

5.12 $$r_{1}+r_{2}=0.$$

The condition (5.12) can be viewed as a consequence of stipulating that

5.13 $$r_{1}^{j}+r_{2}^{j}=0,\;j=1,\ldots M,$$

which in turn amounts to the requirement that the driving forces $f_{j}$, j=1,…,M, defined in (5.4) involve $\mu_{1}$ and $\mu_{2}$ through only their difference $\mu_{2}-\mu_{1}$. This also requires that $\beta_{1}^{j}-\alpha_{1}^{j}=-(\beta_{2}^{j}-\alpha_{2}^{j})$. By the same reasoning, $f_{j}$, j=1,…,M, can involve $\mu_{N-1}$ and $\mu_{N}$ only through their difference $\mu_{N-1}-\mu_{N}$. Moreover, the condition $\beta_{N}^{j}-\alpha_{N}^{j}=-(\beta_{N-1}^{j}-\alpha_{N-1}^{j})$ must be satisfied. The aforementioned conditions on the stoichiometric coefficients can be conveniently summarized as follows:

5.14 $$\beta_{1}^{j}+\beta_{2}^{j}=\alpha_{1}^{j}+\alpha_{2}^{j}\;\text{and}\;\beta_{N}^{j}+\beta_{N-1}^{j}=\alpha_{N}^{j}+\alpha_{N-1}^{j},\;j=1,\ldots ,M.$$

As explained in the electronic supplementary material, by applying the conventional treatment of internal constraints in continuum mechanics to the constraints (5.10) and (5.11), it can be deduced that $\mu_{I}$ and $\zeta_{I}$, with I∈{1,2,N−1,N}, have the forms:

5.15 $$\mu_{I}=\mu_{I}^{r}+\mu_{I}^{a}\;\text{and}\;\zeta_{I}=\zeta_{I}^{r}+\zeta_{I}^{a},\;I\in \{1,2,N-1,N\},$$

where $q^{\text{r}}$ and $q^{\text{a}}$ denote the reactive and active parts of a generic quantity q, respectively. Also,

5.16 $$\begin{array}{cc} \mu_{1}^{r}=\mu_{2}^{r}=:\omega , & \zeta_{1}^{r}=\zeta_{2}^{r}=:\omega ,\\ \mu_{N-1}^{r}=\mu_{N}^{r}=:\varpi , & \zeta_{N-1}^{r}=\zeta_{N}^{r}=:\varpi . \end{array}\}$$

Notice that, by (5.16)${\;}_{1,2}$,

5.17 $$\begin{array}{cc} \mu_{21}:=\mu_{2}-\mu_{1}=\mu_{2}^{a}-\mu_{1}^{a}, & \zeta_{21}:=\zeta_{2}-\zeta_{1}=\zeta_{2}^{a}-\zeta_{1}^{a},\\ \mu_{(N-1)N}:=\mu_{N-1}-\mu_{N}=\mu_{N-1}^{a}-\mu_{N}^{a}, & \zeta_{(N-1)N}:=\zeta_{N-1}-\zeta_{N}=\zeta_{N-1}^{a}-\zeta_{N}^{a}. \end{array}\}$$

The condition $f_{j}=0$, with j∈1,…,M, can be treated as an internal constraint with associated Lagrange multiplier being the reaction rate $r^{j}$. Suppose, for example, that

5.18 $$f_{1}=\mu_{B}+\mu_{1}-\mu_{2}=0\;\;⟺\;\;\mu_{B}=\mu_{2}-\mu_{1},$$

whereby the adsorption reaction involving the bulk constituent B occurs very rapidly relative to all other reactions and, consequently, local chemical equilibrium is achieved instantaneously in bulk. Thus, rather than being determined by a constitutive equation, the reaction rate $r^{1}$ is a Lagrange multiplier associated with the constraint (5.18).

To conclude this subsection, we emphasize that precedents for the decomposition (5.15)${\;}_{1}$ of the chemical potential can be found in works on incompressible binary polymer mixtures by de Gennes [10] and by E & Palffy–Muhoray [11]. de Gennes postulated the existence of an extra, repulsive, contribution to the chemical potential that is analogous to our reactive contribution $\mu_{I}^{r}$, whereas E & Palffy–Muhoray derived a corresponding contribution by considering the incompressible limit of an unconstrained theory.

### (b) Constitutive equations

We next formulate constitutive equations. Guided by the inequality

5.19 $${\dot{\psi }}_{S}-\sum_{I=3}^{N-2}(\mu_{I}{\dot{n}}_{I}-\zeta_{I}\cdot {ȷ}_{I})-\mu_{21}{\dot{n}}_{2}-\mu_{(N-1)N}{\dot{n}}_{N-1})-\zeta_{21}\cdot {ȷ}_{2}-\zeta_{N-1}\cdot {ȷ}_{N-1}-\sum_{j=1}^{M}f_{j}r^{j}\leq 0,$$

arising from using (5.10), (5.11) and (5.17) in (5.3), we choose

5.20 $$s:=(n,j,r)=(\underset{n}{\underbrace{n_{2},n_{3},\ldots ,n_{N-1}}},\underset{j}{\underbrace{{ȷ}_{2},\ldots ,{ȷ}_{N-1}}},\underset{r}{\underbrace{r^{1},\ldots ,r^{M}}})$$

and

5.21 $$q:=(\psi_{S},\mu_{21},\mu_{3},\ldots ,\mu_{N-2},\mu_{(N-1)N},\zeta_{21},\zeta_{3},\ldots ,\zeta_{N-2},\zeta_{(N-1)N},f_{1},\ldots ,f_{M}),$$

as independent and dependent variables, respectively, and, consequently, introduce the constitutive equations of the type

5.22 $$q=\hat{q}(s),$$

for each q in the list q.

By combining (5.19) and (5.22) with the provision that for a fixed s the corresponding rate $\dot{s}$ can be chosen arbitrarily, we invoke the procedure of Coleman & Noll [12] to find that:

(i) $\psi_{S}$ must be composition dependent at most and thus given by a relation of the form
  5.23 $$\psi_{S}={\hat{\psi }}_{S}(n);$$
(ii) $\mu_{21}$, $\mu_{(N-1)N}$ and $\mu_{I}$, with I=3,…,N−2, must be generated from the derivative of the response function ${\hat{\psi }}_{S}$ through the relations
  5.24 $$\mu_{21}=\frac{\partial {\hat{\psi }}_{S}(n)}{\partial n_{2}},\;\mu_{(N-1)N}=\frac{\partial {\hat{\psi }}_{S}(n)}{\partial n_{N-1}},\;\mu_{I}=\frac{\partial {\hat{\psi }}_{S}(n)}{\partial n_{I}},\;I=3,\ldots ,N-2;$$
(iii) the response functions ${\hat{\zeta }}_{21}$, ${\hat{\zeta }}_{(N-1)N}$, ${\hat{\zeta }}_{I}$, I=3,…,N−2, and ${\hat{f}}_{j}$, j=1,…,M, must satisfy the residual free-energy inequality
  5.25 $${\hat{\zeta }}_{21}(s)\cdot {ȷ}_{2}+{\hat{\zeta }}_{(N-1)N}(s)\cdot {ȷ}_{N-1}+\sum_{I=3}^{N-2}{\hat{\zeta }}_{I}(s)\cdot {ȷ}_{I}-\sum_{j=1}^{M}{\hat{f}}_{j}(s)\cdot r^{j}\leq 0,$$
  for all admissible s. If all dissipative processes are uncoupled, (5.25) reduces to
  5.26 $$\begin{array}{rl} {\hat{\zeta }}_{21}(n,{ȷ}_{2})\cdot {ȷ}_{2}\leq 0\; & \text{for all }{ȷ}_{2},\\ {\hat{\zeta }}_{(N-1)N}(n,{ȷ}_{N-1})\cdot {ȷ}_{N-1}\leq 0\; & \text{for all }{ȷ}_{N-1},\\ {\hat{\zeta }}_{I}(n,{ȷ}_{I})\cdot {ȷ}_{I}\leq 0\; & \text{for all }{ȷ}_{I},I=3,\ldots ,N-2,\\ {\hat{f}}_{j}(n,r^{j})\cdot r^{j}\geq 0\; & \text{for all }r^{j},j=1,\ldots ,M. \end{array}\}$$

#### (i) The active part of the chemical potentials

Due to the foregoing constitutive developments, the chemical potentials $\mu_{1}$ and $\mu_{2}$ (respectively, $\mu_{N-1}$ and $\mu_{N}$) of constituent $A_{1}$ and $A_{2}$ (respectively, $A_{N-1}$ and $A_{N}$) are not required individually since only their difference $\mu_{2}-\mu_{1}$ (respectively, $\mu_{N-1}-\mu_{N}$) appears in the analysis. However, as will later become clear, the corresponding active parts are central to the description of the reaction kinetics. Hence, we now discuss the prescription of constitutive equations for $\mu_{J}^{\text{a}}$, the active part of $\mu_{J}$, J∈{1,2,N−1,N}. These equations must comply with (5.17) and (5.24)${\;}_{1,2}$.

Towards the expressed objective, we suppose that $\psi_{S}$ can be naturally written as a function of $\left(n_{1},n_{2},\ldots ,n_{N-1},n_{N}\right)$, in which case

5.27 $$\psi_{S}={\tilde{\psi }}_{S}(n_{1},n_{2},\ldots ,n_{N-1},n_{N}).$$

The response function ${\hat{\psi }}_{S}$ is obtained from ${\tilde{\psi }}_{S}$ by

5.28 $${\hat{\psi }}_{S}(n_{2},\ldots ,n_{N-1})={\tilde{\psi }}_{S}(n_{1},n_{2},\ldots ,n_{N-1},n_{N}){|}_{n_{1}=\bar{n}-n_{2},\;n_{N}=\bar{\bar{n}}-n_{N-1}}.$$

Granted the representation (5.27) for $\psi_{S}$, we then assign

5.29 $$\mu_{J}^{a}=\frac{\partial {\tilde{\psi }}_{S}(n_{1},n_{2},\ldots ,n_{N-1},n_{N})}{\partial n_{J}}{|}_{n_{1}=\bar{n}-n_{2},\;n_{N}=\bar{\bar{n}}-n_{N-1}},$$

as the constitutive relation determining the active part of $\mu_{J}$, J=1, 2, N−1, and N. In view of (5.28), we conclude that the choice (5.29) is legitimate since it is consistent with (5.17) and (5.24)${\;}_{1,2}$. Recalling that $\mu_{I}^{\text{a}}=\mu_{I}$ for I=3,…,N−2, (5.24)${\;}_{3}$ and (5.28)–(5.29) allow us to write

5.30 $$\mu_{I}^{a}=\frac{\partial {\tilde{\psi }}_{S}(n_{1},n_{2},\ldots ,n_{N-1},n_{N})}{\partial n_{I}}{|}_{n_{1}=\bar{n}-n_{2},\;n_{N}=\bar{\bar{n}}-n_{N-1}},\;I=1,\ldots ,N.$$

The active, or constitutive, part of the chemical potential of constituent $A_{I}$, I=1,…,N, is thus fully determined by the response function ${\tilde{\psi }}_{S}$.

In view of (5.13) and (5.29), we can recast the affinity of the jth reaction (5.4) as follows:

5.31 $$f_{j}=\sum_{I=0}^{N+1}(\alpha_{I}^{j}-\beta_{I}^{j})\mu_{I}^{a},$$

with the provision

5.32 $$\mu_{I}=\mu_{I}^{a}\;\text{for }I=0\text{ and }I=N+1.$$

The foregoing assumption is consistent with the observation that, by (4.2), the chemical potential of the bulk constituent is fully determined by a constitutive equation and the premise that a similar situation holds for the environmental constituent.

For later reference, we rewrite (5.31) as follows:

5.33 $$f_{j}=f_{j}^{+}-f_{j}^{-},\;\text{with}\;f_{j}^{+}=\sum_{I=0}^{N+1}\alpha_{I}^{j}\mu_{I}^{a}\;\text{and}\;f_{j}^{-}=\sum_{I=0}^{N+1}\beta_{I}^{j}\mu_{I}^{a}.$$

The quantities $f_{j}^{+}$ and $f_{j}^{-}$ introduced in (5.33) are called the forward and backward affinities. If $\mu_{I}$ is determined by $n_{I}$ for I=0 and I=N+1, it follows from (5.30) that $f_{j}^{+}$, $f_{j}^{-}$ and $f_{j}$ are functions of n, meaning that $f_{j}^{+}={\hat{f}}_{j}^{+}(n)$, $f_{j}^{-}={\hat{f}}_{j}^{-}(n)$ and $f_{j}={\hat{f}}_{j}(n)$, respectively.

#### (ii) The reaction rates and Marcelin–De Donder kinetics

To discuss the specification of the response function ${\hat{f}}_{j}(n,r^{j}),\;j=1,\ldots ,M$, we begin by assuming that the relation $f_{j}={\hat{f}}_{j}(n,r^{j})$ can be inverted and replaced by

5.34 $$r^{j}={\hat{r}}^{j}(n,f_{j}),$$

where ${\hat{r}}^{j}(n,\cdot )$ denotes the inverse of ${\hat{f}}_{j}(n,\cdot )$, and we consider a response function ${\hat{r}}^{j}(n,f_{j})$ such that

5.35 $$r^{j}={\bar{r}}^{j}(n):={\hat{r}}^{j}(n,{\hat{f}}_{j}(n))=D_{j}(\exp(\frac{{\hat{f}}_{j}^{+}(n)}{k_{B}T})-\exp(\frac{{\hat{f}}_{j}^{-}(n)}{k_{B}T})),\;j=1,\ldots ,M,$$

where $D_{j}$ is a positive constant, $k_{B}$ is the Boltzmann constant and T is the absolute temperature, a choice that is consistent with (5.26)${\;}_{4}$. In this case, the jth reaction is said to display kinetics of the Marcelin–De Donder type (see, for instance, Marcelin [13], De Donder [14], Van Rysselberghe [15] and Feinberg [16]).

We can express the relation (5.35) for $r^{j}$ in a different form by introducing the reaction rates $r_{f}^{j}$ and $r_{B}^{j}$ associated with the forward and backward parts of the jth reaction and by defining the activity $a_{I}:=\exp((\mu_{I}^{\text{a}}-\mu_{I}^{0})/k_{B}T)$ of constituent $A_{I}$, I∈{0,…,N+1}, where $\mu_{I}^{0}$ denotes the active part of the chemical potential of $A_{I}$ at $a_{I}=1$. Accordingly, by (5.33), the relation can be replaced by

5.36 $$r^{j}=r_{f}^{j}-r_{B}^{j},\;\text{with}\;r_{f}^{j}=k_{j}^{+}\prod_{I=0}^{N+1}a_{I}^{\alpha_{I}^{j}}\;\text{and}\;r_{B}^{j}=k_{j}^{-}\prod_{I=0}^{N+1}a_{I}^{\beta_{I}^{j}}.$$

Here and henceforth, $k_{j}^{+}$ and $k_{j}^{-}$ defined by

5.37 $$\ln(\frac{k_{j}^{+}}{D_{j}})=\frac{1}{k_{B}T}\sum_{I=0}^{N+1}\alpha_{I}^{j}\mu_{I}^{0}\;\text{and}\;\ln(\frac{k_{j}^{-}}{D_{j}})=\frac{1}{k_{B}T}\sum_{I=0}^{N+1}\beta_{I}^{j}\mu_{I}^{0},\;j=1,\ldots ,M$$

are the constant rates of the forward and backward parts of the jth reaction. A consequence of (5.37) is that those rates of reaction must be related by

5.38 $$\frac{k_{j}^{+}}{k_{j}^{-}}=\exp(\frac{1}{k_{B}T}\sum_{I=0}^{N+1}(\alpha_{I}^{j}-\beta_{I}^{j})\mu_{I}^{0}).$$

Relations (5.36) embody the requirement that the forward and backward reaction rates must be proportional to the product of the activity of the corresponding reactants raised to the corresponding forward and backward stoichiometric coefficients. With (5.38), the relations (5.36) generalize the classical law of mass action kinetics as applied to the jth reaction (see, for instance, Gorban [17]). Indeed, if the activity of each constituent $A_{I}$ that is present is set to $a_{I}=c_{I}=n_{I}/{\bar{n}}_{I}$, which amounts to taking that the active part $\mu_{I}^{a}$ of the chemical potential of $A_{I}$ to be given by

5.39 $$\mu_{I}^{a}=\mu_{I}^{0}+k_{B}T\ln\frac{n_{I}}{{\bar{n}}_{I}},$$

where ${\bar{n}}_{I}$ is the reference value for the number density of constituent $A_{I}$, then (5.36) yields the classical law of mass-action kinetics. With this specialization, the reaction rate $r^{j}$ depends only on the concentrations of the reactants and their stoichiometric coefficients, as given classically.

## 6. Application

We suppose hereinafter that ∂R contains two independent lattices, one occupied by constituent A, which is adsorbed from the bulk, and the other occupied by constituent C, which may or may not be adsorbed from the environment. In so doing, we introduce constituents $A^{*}$ and $C^{*}$ that account for vacant sites on the lattices for constituents A and C, respectively. The total number N of surface constituents is therefore N=4, and the chemical system under consideration involves constituents $A_{0}=B$, $A_{1}=A^{*}$, $A_{2}=A$, $A_{3}=C$, $A_{4}=C^{*}$ and $A_{5}=E$.

For the system under consideration and in absence of constituent supplies, the governing equations (3.5) specialize to

6.1 $$\begin{array}{l} {\dot{n}}_{B}=-\text{ div}\;{ȷ}_{B}\;\text{on}\;R;\\ \begin{array}{l} {ȷ}_{B}\cdot n+r_{B}=0,\\ {\dot{n}}_{A}=-{\text{ div}}_{\;S\;}{ȷ}_{A}+r_{A},\\ {\dot{n}}_{C}=-{\text{ div}}_{\;S\;}{ȷ}_{C}+r_{C},\\ -{ȷ}_{E}\cdot n+r_{E}=0, \end{array}\}\;\text{on}\;\partial R. \end{array}\}$$

In writing (6.1), we have omitted the equations for the vacancy constituents $A^{*}$ and $C^{*}$ in view of the constraints

6.2 $$\begin{array}{c} n_{A}+n_{A^{*}}={\bar{n}}_{A},\\ {ȷ}_{A}+{ȷ}_{A^{*}}=0,\\ r_{A}+r_{A^{*}}=0, \end{array}\}\;\text{and}\;\begin{array}{c} n_{C}+n_{C^{*}}={\bar{n}}_{C},\\ {ȷ}_{C}+{ȷ}_{C^{*}}=0,\\ r_{C}+r_{C^{*}}=0, \end{array}\}$$

in which the number density of lattice sites ${\bar{n}}_{A}$ and ${\bar{n}}_{C}$ are given constants.

### (a) Reaction mechanism

The constituents $A_{0}$, $A_{1}$, …, $A_{5}$, are involved in the following elementary reactions:

6.3 $$\begin{array}{rl} & \;(R_{1}):\;A_{0}+A_{1}⇌A_{2},\\ & \;(R_{2}):\;A_{1}+2A_{2}⇌3A_{2},\\ & \;(R_{3}):\;2A_{1}+A_{2}⇌3A_{1},\\ & \;(R_{4}):\;A_{1}+A_{3}⇌A_{2}+A_{3},\\ & \;(R_{5}):\;A_{2}+A_{3}⇌A_{2}+A_{4},\\ & \;(R_{6}):\;A_{2}+A_{4}⇌A_{1}+A_{4},\\ & \;(R_{7}):\;A_{1}+A_{4}⇌A_{1}+A_{3},\\ & \;(R_{8}):\;A_{5}+A_{4}⇌A_{3}. \end{array}\}$$

While the reaction $R_{1}$ (respectively $R_{8}$) corresponds to the adsorption of constituents B (respectively E) by ∂R, the remaining reactions $R_{2}$–$R_{7}$ were introduced by Malevanets & Kapral [4] to provide a chemical scheme with kinetics of the FitzHugh–Nagumo type. They viewed that scheme as a means for describing systems that are far from equilibrium and, hence, assumed that the system is subject to external flows of reagents that are not explicitly included in the reaction scheme. They took the concentrations of these feed constituents to be constant and incorporated those concentrations in the rate constants. Furthermore, they took all reactions to be irreversible in the sense that they proceed solely from left to right. This amounts to assuming that $r_{f}^{j}\gg r_{B}^{j}$ for j≠1 and j≠8.

On inspection of (6.3), the non-null stoichiometric coefficients can be identified and used in (5.2) and (5.4) to deliver the production rates and affinities in terms of the reaction rates and the chemical potentials, respectively.

### (b) Constitutive specialization

We consider a particular theory based on the following constitutive choices for the response functions determining the bulk and surface free-energy densities $\hat{\psi }$, ${\tilde{\psi }}_{S}$ and ${\hat{\psi }}_{S}$:

6.4 $$\begin{array}{rl} & \hat{\psi }(n_{B})=\mu_{B}^{0}n_{B}+k_{B}Tn_{B}(\ln\frac{n_{B}}{{\bar{n}}_{B}}-1),\\ & {\tilde{\psi }}_{S}(n_{A},n_{A^{*}},n_{C},n_{C^{*}})=\sum_{X\in C}n_{X}(\mu_{X}^{0}+k_{B}T\ln\frac{n_{X}}{{\bar{n}}_{X}}),\\ & {\hat{\psi }}_{S}(n_{A},n_{C})={\tilde{\psi }}_{S}(n_{A},{\bar{n}}_{A}-n_{A},n_{C},{\bar{n}}_{C}-n_{C}), \end{array}\}$$

where $\mu_{B}^{0}$ is the chemical potential of constituent B at density ${\bar{n}}_{B}$ and where $\mu_{X}^{0}$ is the chemical potential of constituent X at density $n_{X}={\bar{n}}_{X}$. Recalling that ${\bar{n}}_{A}={\bar{n}}_{A^{*}}=\bar{n}$ and ${\bar{n}}_{C}={\bar{n}}_{C^{*}}=\bar{\bar{n}}$, we can use (4.2) and (5.24) to give

6.5 $$\begin{array}{rl} & \mu_{B}=\mu_{B}^{0}+k_{B}T\ln\frac{n_{B}}{{\bar{n}}_{B}},\\ & \mu_{21}=\mu_{A}^{0}-\mu_{A^{*}}^{0}+k_{B}T\ln\frac{n_{A}}{{\bar{n}}_{A}-n_{A}},\\ & \mu_{34}=\mu_{C}^{0}-\mu_{C^{*}}^{0}+k_{B}T\ln\frac{n_{C}}{{\bar{n}}_{C}-n_{C}}, \end{array}\}$$

from which we conclude that

6.6 $$\begin{array}{rl} & f_{1}=\mu_{B}^{0}-\mu_{A}^{0}+\mu_{A*}^{0}+k_{B}T\ln\frac{({\bar{n}}_{A}-n_{A})n_{B}}{n_{A}{\bar{n}}_{B}},\\ & f_{8}=\mu_{E}-\mu_{0}^{C}-k_{B}T\ln\frac{n_{C}}{{\bar{n}}_{C}-n_{C}}. \end{array}\}$$

We now specify the reaction rates $r^{j}$ for j=1,…,8. Specifically, observing from (5.30) and (6.4)${\;}_{2}$ that the expressions for the active parts of the chemical potentials of constituent A, $A^{*}$, C and $C^{*}$ have the form (5.39) and assuming that the chemical potential of the external constituent has this same form, we use (5.36) to infer that

6.7 $$\begin{array}{rl} & r^{1}=k_{1}^{+}b(1-a)-k_{1}^{-}a,\;r^{2}=k_{2}^{+}a^{2}(1-a)-k_{2}^{-}a^{3},\\ & r^{3}=k_{3}^{+}{\left(1-a\right)}^{2}c-k_{3}^{-}{\left(1-a\right)}^{3},\;r^{4}=k_{4}^{+}(1-a)c-k_{4}^{-}ac,\\ & r^{5}=k_{5}^{+}ac-k_{5}^{-}a(1-c),\;r^{6}=k_{6}^{+}a(1-c)-k_{6}^{-}(1-a)(1-c),\\ & r^{7}=k_{7}^{+}(1-a)(1-c)-k_{7}^{-}(1-a)c,\;r^{8}=k_{8}^{+}e(1-c)-k_{8}^{-}c, \end{array}\}$$

where $k_{j}^{\pm }$, j=1,…,8, are the reaction constants and where b, a, c and e are the dimensionless constituent concentrations defined according to

6.8 $$b:=\frac{n_{B}}{{\bar{n}}_{B}},\;a:=\frac{n_{A}}{{\bar{n}}_{A}},\;c:=\frac{n_{C}}{{\bar{n}}_{C}}\;\text{and}\;e:=\frac{n_{E}}{{\bar{n}}_{E}}.$$

In the absence of feed constituents, the reaction constants would be obtained by using (5.37). Notice that we also have invoked the lattice constraints (5.10)${\;}_{1}$ and (5.10)${\;}_{2}$ to write $1-a=n_{A^{*}}/{\bar{n}}_{A}$ and $1-c=n_{C^{*}}/{\bar{n}}_{C}$.

We assume that the response functions ${\hat{\zeta }}_{B}$, ${\hat{\zeta }}_{A}:={\hat{\zeta }}_{21}$ and ${\hat{\zeta }}_{C}:={\hat{\zeta }}_{43}$ are given by

6.9 $$\begin{array}{rl} & {\hat{\zeta }}_{B}(n_{B},{ȷ}_{B})=-\frac{k_{B}T}{D_{B}n_{B}}{ȷ}_{B},\\ & {\hat{\zeta }}_{A}(n_{A},n_{C},{ȷ}_{A})=-\frac{{\bar{n}}_{A}k_{B}T}{D_{A}^{S}n_{A}({\bar{n}}_{A}-n_{A})}{ȷ}_{A},\\ & {\hat{\zeta }}_{C}(n_{A},n_{C},{ȷ}_{B})=-\frac{{\bar{n}}_{C}k_{B}T}{D_{C}^{S}n_{C}({\bar{n}}_{C}-n_{C})}{ȷ}_{C}, \end{array}\}$$

where $D_{B}>0$, $D_{A}^{S}>0$ and $D_{C}^{S}>0$ are the diffusivities of constituents B, A and C, respectively. With reference to (6.5), we see that (6.9) furnishes the expressions,

6.10 $${ȷ}_{B}=-D_{B}\nabla n_{B},\;{ȷ}_{A}=-D_{A}^{S}\nabla_{\;S}\;n_{A}\;\text{and}\;{ȷ}_{C}=-D_{C}^{S}\nabla_{\;S}\;n_{C},$$

for the fluxes ${ȷ}_{B}$, ${ȷ}_{A}$ and ${ȷ}_{C}$.

If we assume that the corresponding driving force $f_{1}$ for the sorption reaction (5.1) vanishes identically, then it follows from (6.6)${\;}_{1}$ and (6.8) that

6.11 $$a=\frac{Kb}{1+Kb},\;\text{with}\;K=\exp\frac{\mu_{B}^{0}+\mu_{A^{*}}^{0}-\mu_{A}^{0}}{k_{B}T}.$$

In this case, $r^{1}$ must be viewed a ‘reactive force’ corresponding to the constraint $f_{1}=0$.

#### (i) Production rates

The production rates $r_{B}$, $r_{A}$, $r_{C}$ and $r_{E}$ of constituents B, A, C and E can be written in terms of the reaction rates $r^{j}$, j=1,…,8, as follows:

6.12 $$r_{B}=-r^{1},\;r_{A}=r^{1}+r^{2}+r^{4}-r^{3}-r^{6},\;r_{C}=r^{7}+r^{8}-r^{5},\;r_{E}=-r^{8}.$$

Moreover, $r_{B}$ and $r_{E}$ are related to ${ȷ}_{B}\cdot n$ and ${ȷ}_{E}\cdot n$ by (3.2) and (3.4), respectively. On using (6.7)${\;}_{2-7}$, the production rates $r_{A}$ and $r_{C}$ thus become

6.13 $$r_{A}={ȷ}_{B}\cdot n+F^{+}(a,c)+F^{-}(a,c)\;\text{and}\;r_{C}={ȷ}_{E}\cdot n+G^{+}(a,c)+G^{-}(a,c),$$

where ${ȷ}_{B}\cdot n$ and ${ȷ}_{E}\cdot n$ account for the sorption processes involving the bulk and environmental constituent B and E, respectively, and the coefficients $F^{\pm }$ and $G^{\pm }$ are defined according to

6.14 $$\begin{array}{rl} & F^{+}(a,c)=k_{2}^{+}a^{2}(1-a)+k_{4}^{+}(1-a)c-k_{3}^{+}{\left(1-a\right)}^{2}a-k_{6}^{+}a(1-c),\\ & F^{-}(a,c)=k_{3}^{-}(1-a)+k_{6}^{-}(1-a)(1-c)-k_{2}^{-}a^{3}-k_{4}^{-}ac,\\ & G^{+}(a,c)=k_{7}^{+}(1-a)(1-c)-k_{5}^{+}ac,\\ & G^{-}(a,c)=k_{5}^{-}(1-a)c-k_{7}^{-}(1-a)c. \end{array}\}$$

#### (ii) Sorption processes

The present work focuses on the following possibilities for describing the sorption reaction involving the bulk constituent B, possibilities which lead to different boundary conditions for the bulk diffusion problem:

— The reaction rate is given by (6.7)${\;}_{1}$ and hence, by (3.2) and (6.12)${\;}_{1}$, takes the form
  6.15 $${ȷ}_{B}\cdot n=k_{1}^{+}b(1-a)-k_{1}^{-}a.$$
— The sorption reaction is so rapid that $f_{1}=0$ and, hence, (6.11) holds, i.e. the relation
  6.16 $$a=\frac{Kb}{1+Kb}$$
  between the concentrations $b=n_{B}/{\bar{n}}_{B}$ and $a=n_{A}/{\bar{n}}_{A}$ therefore prevails. The relation (6.16) corresponds to the Langmuir isotherm (see, for instance, Houston [8]).

As for the sorption reaction involving the environmental constituent E, we may consider that its rate is given by (6.7)${\;}_{8}$ and hence, by (3.4) and (6.12)${\;}_{4}$, that

6.17 $${ȷ}_{E}:={ȷ}_{E}\cdot n=k_{8}^{-}c-k_{8}^{+}e(1-c).$$

In the absence of sorption, $r^{8}=0$ and, thus, by (3.4), ${ȷ}_{E}\cdot n=0$, in which case (6.17) reduces to ${ȷ}_{E}=0$.

### (c) Governing equations

The governing equations of the theory are obtained by combining the content balances for constituents B, A and C with the constitutive theory specified in the previous subsection. If, for instance, we assume that the sorption of the bulk constituent B occurs very rapidly and that ${ȷ}_{E}=0$, we arrive, after combining (6.1), (6.10), (6.13) and (6.16), at a system for the dimensionless concentrations a, b and c of the form

6.18 $$\begin{array}{l} \dot{b}=D_{B}△b\;\text{on}\;R;\\ \begin{array}{l} \frac{Kb}{1+Kb}=a,\\ \dot{a}=D_{A}^{S}{△}_{S}a-\frac{D_{B}{\bar{n}}_{B}}{{\bar{n}}_{A}}\nabla b\cdot n+F(a,c),\\ \dot{c}=D_{C}^{S}{△}_{S}c+G(a,c), \end{array}\}\;\text{on}\;\partial R, \end{array}\}$$

where F and G are defined by

6.19 $$F(a,c)=\frac{1}{{\bar{n}}_{A}}(F^{+}(a,c)+F^{-}(a,c))\;\text{and}\;G(a,c)=\frac{1}{{\bar{n}}_{C}}(G^{+}(a,c)+G^{-}(a,c)),$$

with $F^{\pm }$ and $G^{\pm }$ as given in (6.14). Recall that the contributions $F^{-}$ and $G^{-}$ to F and G can be neglected, in which case the reactions $R_{j}$, j=2,…,7, are assumed to be irreversible.

### (d) Dimensionless equations

Let $L_{C}$ and $t_{C}$ be characteristic measures of length and time, respectively. Granted that each reaction $R_{j}$, j=2,…,7, in (6.3) is irreversible, we may then rewrite the conditions (6.18) in the dimensionless form

6.20 $$\begin{array}{l} \dot{b}=\frac{t_{C}D_{B}}{L_{C}^{2}}△b\;\text{on}\;\Omega ;\\ \begin{array}{l} a=\frac{Kb}{1+Kb},\\ \dot{a}=\frac{t_{C}D_{A}^{S}}{L_{C}^{2}}{△}_{S}a-\frac{t_{C}{\bar{n}}_{B}D_{B}}{{\bar{n}}_{A}L_{C}}\nabla b\cdot n+\bar{F}(a,c),\\ \dot{c}=\frac{t_{C}D_{C}^{S}}{L_{C}^{2}}{△}_{S}c+\bar{G}(a,c), \end{array}\}\;\text{on}\;\partial \Omega , \end{array}\}$$

with

6.21 $$\begin{array}{rl} & \bar{F}(a,c)=t_{C}(k_{1}a^{2}(1-a)-k_{2}a{\left(1-a\right)}^{2}+k_{3}(1-a)c-k_{5}a(1-c)),\\ & \bar{G}(a,c)=t_{C}(k_{6}(1-a)(1-c)-k_{4}ac), \end{array}\}$$

where $k_{i}$, i=1,…,6, are given by

6.22 $$k_{1}=\frac{k_{1}^{+}}{{\bar{n}}_{A}^{4}},\;k_{2}=\frac{k_{2}^{+}}{{\bar{n}}_{A}^{4}},\;k_{3}=\frac{k_{3}^{+}}{{\bar{n}}_{A}^{2}{\bar{n}}_{B}},\;k_{4}=\frac{k_{4}^{+}}{{\bar{n}}_{A}{\bar{n}}_{B}^{2}},\;k_{5}=\frac{k_{5}^{+}}{{\bar{n}}_{A}^{2}{\bar{n}}_{B}},\;k_{6}=\frac{k_{6}^{+}}{{\bar{n}}_{A}{\bar{n}}_{B}^{2}},$$

and where ∇, △ and ${△}_{S}$ now denote dimensionless versions of the originally introduced operators.

### (e) A reaction–diffusion system with FitzHugh–Nagumo kinetics

We now use (6.20) to obtain a reaction–diffusion system in a dimensionless form. Specifically, we

(1) Assume that the dimensionless bulk concentration b is low enough so that Kb≪1 and, hence, that (6.20)${\;}_{2}$ can be replaced by
  6.23 a=Kb.
(2) Assume, following Malevanets & Kapral [4], that $k_{3}=k_{5}$ and $k_{4}=k_{6}$, and introduce the changes of variables
  6.24 $$a=c_{A}u+a_{0}\;\text{and}\;c=c_{C}v+c_{0}$$
  for the surface concentrations a and c, with
  6.25 $$\begin{array}{rl} & c_{A}^{2}=\frac{1}{3}{(\frac{k_{1}+2k_{2}}{k_{1}+k_{2}})}^{2}-\frac{k_{2}+k_{3}}{k_{1}+k_{2}},\;a_{0}=\frac{1}{3}\frac{k_{1}+2k_{2}}{k_{1}+k_{2}},\\ & c_{C}=-\frac{k_{1}+k_{2}}{k_{3}}c_{A}^{3},\;c_{0}=a_{0}c_{C}(1-{(\frac{a_{0}c_{C}}{c_{A}})}^{\;2\;}), \end{array}\}$$
  and stipulate that the characteristic time $t_{C}$ be given by
  6.26 $$t_{C}=\frac{1}{(k_{1}+k_{2})c_{A}^{2}}.$$
(3) Introduce the change of variable
  6.27 $$U=\frac{Kb}{c_{A}}-\frac{a_{0}}{c_{A}},$$
  for the bulk dimensionless concentration b.

With the information introduced in steps 1–3, we find that (6.20) can be replaced by

6.28 $$\begin{array}{l} \dot{U}=\alpha △U\;\text{on}\;\Omega ,\\ \begin{array}{l} u=U,\\ \dot{u}=d_{u}{△}_{S}u-\beta \nabla U\cdot n+f(u,v),\\ \dot{v}=d_{v}{△}_{S}v+g(u,v), \end{array}\}\;\text{on}\;\partial \Omega , \end{array}\}$$

where α, $d_{u}$, $d_{v}$ and β are defined by

6.29 $$\alpha =\frac{t_{C}D_{B}}{L_{C}^{2}},\;d_{u}=\frac{t_{C}D_{A}^{S}}{L_{C}^{2}},\;d_{v}=\frac{t_{C}D_{C}^{S}}{L_{C}^{2}}\;\text{and}\;\beta =\frac{{\bar{n}}_{B}t_{C}D_{B}}{{\bar{n}}_{A}L_{C}K},$$

with $t_{C}$ as given in (6.26), and where f and g are defined by

6.30 $$f(u,v)=-u^{3}+u-v\;\text{and}\;g(u,v)=\epsilon (u-\gamma v-\lambda ),$$

with γ, λ and ϵ given by

6.31 $$\gamma =-\frac{c_{C}}{c_{A}},\;\lambda =\frac{1-a_{0}-c_{0}}{c_{A}},\;\epsilon =\frac{k_{4}}{k_{2}}{(\frac{c_{A}}{c_{C}})}^{\;2}.$$

From the particular forms of f and g, the system of equations (6.28) describes the coupling between bulk diffusion and surface reaction–diffusion with chemical kinetics of the type introduced by FitzHugh [18] and later refined by Nagumo *et al.* [19], as f and g are given by (6.30).

### (f) Linear stability analysis. Instability driven by surface diffusion

We now investigate whether the chemical system described by (6.28) can exhibit diffusion-driven Turing [20] instabilities. Specifically, we explore conditions under which a homogeneous steady state, which is stable to small perturbations in the absence of surface diffusion, becomes unstable to small perturbations in the presence of surface diffusion. Towards this end, we proceed in a manner similar to the classical Turing instability analysis (see, for instance, Murray [21]) and study whether, when subjected to small perturbations, a homogeneous state is stable against spatially homogeneous perturbations of the surface quantities but is unstable against more general perturbations. Our approach follows closely that adopted by Rätz & Röger [22], who analysed a system of equations for cell polarization that is distinct from (6.28). Our main purpose is to investigate the role played by the bulk-surface coupling, as represented by the parameter β on the occurrence of surface diffusion-driven instabilities.

#### (i) Small perturbations of homogeneous steady states

A homogeneous steady state $\left(U_{*},u_{*},v_{*}\right)$ corresponds to a space- and time-independent solution of (6.28) and hence is obtained by solving the steady-state system

6.32 $$u_{*}=U_{*},\;f(u_{*},v_{*})=0,\;g(u_{*},v_{*})=0.$$

We now consider small perturbations $\left(\tilde{U},\tilde{u},\tilde{v}\right)$ of a generic homogeneous steady state $\left(U_{*},u_{*},v_{*}\right)$ determined by solving (6.32). Such perturbations are governed by the system of equations obtained by linearizing (6.28) around $\left(U_{*},u_{*},v_{*}\right)$, which read:

6.33 $$\begin{array}{l} \dot{\tilde{U}}=\alpha △\tilde{U}\;\text{on}\;\Omega ;\\ \begin{array}{l} \tilde{u}=\tilde{U},\\ \dot{\tilde{u}}=d_{u}{△}_{S}\tilde{u}-\beta \nabla \tilde{U}\cdot n+f_{u}\tilde{u}+f_{v}\tilde{v},\\ \dot{\tilde{v}}=d_{v}{△}_{S}\tilde{v}+g_{u}\tilde{u}+g_{v}\tilde{v}, \end{array}\}\;\text{on}\;\partial \Omega . \end{array}\}$$

Here and henceforth, $f_{u}$ and $f_{v}$ denote the partial derivatives of f with respect to u and v evaluated at $\left(u,v)=(u_{*},v_{*}\right)$. Analogously, $g_{u}$ and $g_{v}$ denote the partial derivatives of g with respect to u and v evaluated at $\left(u,v)=(u_{*},v_{*}\right)$. In view of (6.30), we thus have that

6.34 $$f_{u}=1-3u_{*}^{2},\;f_{v}=-1,\;g_{u}=\epsilon \;\text{and}\;g_{v}=-\epsilon \gamma .$$

We now assume that Ω∪∂Ω is a unit ball and, hence, that $\Gamma :=\partial \Omega =S^{2}$ is the unit sphere. Then, following Rätz & Röger [22], we introduce a polar coordinate system in which a point x in Ω∪∂Ω is represented as x=ry, with 0≤r≤1 and y belonging to $S^{2}$, and we introduce an orthonormal basis for $L^{2}(\Gamma )$ consisting of the spherical harmonics $\varphi_{lm}$ defined according to

6.35 $${△}_{S}\varphi_{lm}=-l(l+1)\varphi_{lm}\;\text{on}\;\Gamma ,$$

where the integers l and m are selected to ensure that l≥0 and |m|≤l. We now seek solutions $\left(\tilde{U},\tilde{u},\tilde{v}\right)$ of the linear problem (6.33) of the form

6.36 $$\begin{array}{rl} & \tilde{U}(ry,t)=\sum_{l=0}^{\infty }\sum_{m=-l}^{l}{\bar{U}}_{lm}e^{\omega t}\psi_{lm}(r)\varphi_{lm}(y),\\ & \tilde{u}(y,t)=\sum_{l=0}^{\infty }\sum_{m=-l}^{l}{\bar{u}}_{lm}e^{\omega t}\varphi_{lm}(y),\\ & \tilde{v}(y,t)=\sum_{l=0}^{\infty }\sum_{m=-l}^{l}{\bar{v}}_{lm}e^{\omega t}\varphi_{lm}(y), \end{array}\}$$

where the amplitudes ${\bar{U}}_{lm}$, ${\bar{u}}_{lm}$ and ${\bar{v}}_{lm}$ are constant. Substituting the Ansatz (6.36) into (6.33), cancelling the common factor $e^{\omega t}$ and computing the $L^{2}(\Gamma )$ scalar product of the resulting equations with $\varphi_{lm}$, we find that, for each l and m,

6.37 $$\begin{array}{rl} & r^{2}\psi_{lm}^{\prime \prime }(r)+2r\psi_{lm}^{\prime }(r)-(l(l+1)+\frac{\omega }{\alpha }r^{2})\psi_{lm}(r)=0,\\ & {\bar{U}}_{lm}\psi_{lm}(1)-{\bar{u}}_{lm}=0,\\ & \omega {\bar{u}}_{lm}=-d_{u}l(l+1){\bar{u}}_{lm}+f_{u}{\bar{u}}_{lm}+f_{v}{\bar{v}}_{lm}-\beta \psi_{lm}^{\prime }(1){\bar{U}}_{lm},\\ & \omega {\bar{v}}_{lm}=-d_{v}l(l+1){\bar{v}}_{lm}+g_{u}{\bar{u}}_{lm}+g_{v}{\bar{v}}_{lm}. \end{array}\}$$

We now seek for a condition under which the system comprised by (6.37)${\;}_{2}$–(6.37)${\;}_{4}$ admits non-trivial solutions for $\left({\bar{U}}_{lm},{\bar{u}}_{lm},{\bar{v}}_{lm}\right)$ given that $\psi_{lm}$ is solution of (6.37)${\;}_{1}$. Towards this objective, we begin by noticing that after the change of variable $x=\sqrt{\omega /\alpha }\;r$, it follows from (6.37)${\;}_{1}$ that $\psi_{lm}$, as a function of x, is a solution of the modified spherical Bessel differential equation and hence can be written as a linear combination of the modified spherical Bessel functions of the first and second kinds (see Abramowitz & Stegun [23, §10.2]). However, the presence of the modified spherical Bessel function of the second kind is ruled out in view of its singularity at r=0. Thus,

6.38 $$\psi_{lm}(r)=\alpha_{lm}i_{l}(\sqrt{\frac{\omega }{\alpha }}r),$$

with $\alpha_{lm}\neq 0$ constant and where $i_{l}$ is the modified spherical Bessel function of first kind and order l, which in turn satisfies the relation

6.39 $$i_{l}(x)=\sqrt{\frac{\pi }{2x}}I_{l+\frac{1}{2}}(x),$$

where $I_{\nu }$ is the modified Bessel function of first kind of order ν. The remaining system comprised by (6.37)${\;}_{2-4}$ admits non-trivial solutions for $\left({\bar{U}}_{lm},{\bar{u}}_{lm},{\bar{v}}_{lm}\right)$ if and only if the (dimensionless) growth rate ω is a root of the equation

6.40 $$i_{l}(\sqrt{\frac{\omega }{\alpha }})((\omega -f_{u}^{l})(\omega -g_{v}^{l})-f_{v}g_{u}))+\beta \sqrt{\frac{\omega }{\alpha }}i_{l}^{\prime }(\sqrt{\frac{\omega }{\alpha }})(\omega -g_{v}^{l})=0,$$

where $f_{u}^{l}$ and $g_{v}^{l}$ are defined by

6.41 $$f_{u}^{l}:=f_{u}-d_{u}l(l+1)\;\text{and}\;g_{v}^{l}:=g_{v}-d_{v}l(l+1).$$

A steady state $\left(U_{*},u_{*},v_{*}\right)$ is linearly stable if, for any choice of l, any solution $\omega_{l}$ of (6.40) is such that Re(ωl)<0. Here, we investigate conditions under which a steady state is linearly stable in the absence of surface diffusion, Re(ω0)<0 and linearly unstable if Re(ωl)>0 for some l>0. Together, these conditions characterize diffusion-driven instability.

We observe, for later reference, that for real and positive ω, we can replace (6.40) by

6.42 $$G_{l}(\omega ):=(\omega -f_{u}^{l})(\omega -g_{v}^{l})-f_{v}g_{u}+\kappa_{l}(\omega )(\omega -g_{v}^{l})=0,$$

in which $\kappa_{l}$ is defined by

6.43 $$\kappa_{l}(\omega ):={\beta (\frac{xi_{l}^{\prime }(x)}{i_{l}(x)})|}_{x=\sqrt{w/\alpha }}.$$

In the sequel, we take ω to be real a number.

#### (ii) Stability with respect to spatially homogeneous perturbations

We now consider perturbations $\left(\tilde{U},\tilde{u},\tilde{v}\right)$ of the steady state $\left(U_{*},u_{*},v_{*}\right)$ such that $\tilde{u}$ and $\tilde{v}$ are spatially homogeneous. This requires that ${\bar{u}}_{lm}={\bar{v}}_{lm}=0$ for all l>0. Thus, the steady state $\left(U_{*},u_{*},v_{*}\right)$ is linearly stable if and only if the equation

6.44 $$G_{0}(\omega )=\omega^{2}-(f_{u}+g_{v})\omega +f_{u}g_{v}-f_{v}g_{u}+\kappa_{0}(\omega )(\omega -g_{v})=0,$$

deriving from (6.42) for l=0 has no positive roots.

It can be shown that steady state $\left(U_{*},u_{*},v_{*}\right)$ is linearly stable with respect to spatially homogeneous perturbations if and only if one of the following cases applies:

6.45 $$\text{Case 1:}\;f_{u}+g_{v}\leq 0\;\text{and}\;f_{u}g_{v}-f_{v}g_{u}\geq 0$$

and

6.46 $$\text{Case 2}:\;f_{u}+g_{v}>0\;\text{and}\;\min_{0<\omega <\infty }G_{0}(\omega )=G_{0}(\omega_{0})>0,$$

**Figure 1.**
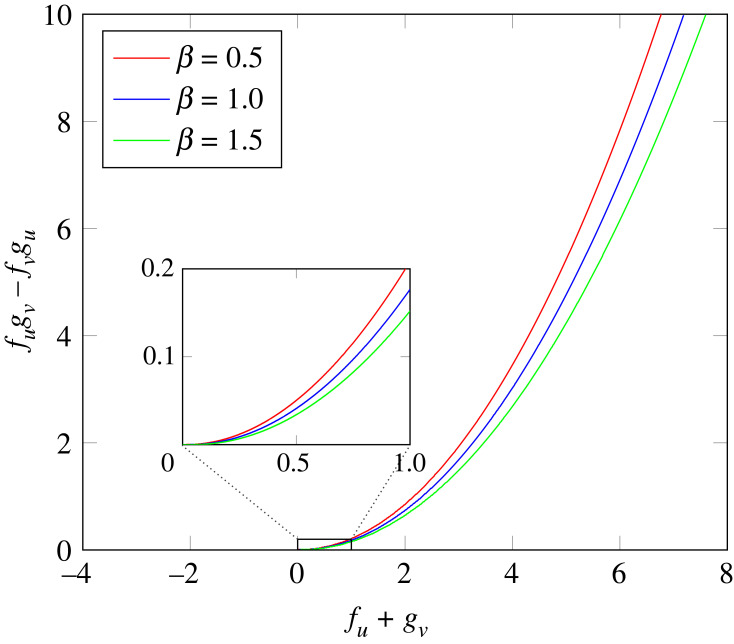
$\left(f_{u}+g_{v},f_{u}g_{v}-f_{v}g_{u}\right)$–diagram for $g_{v}=-0.1$ and different values of β. For each β, the upper (lower) part of the diagram corresponds to homogeneous states that are linearly stable (unstable) with respect to spatially homogeneous perturbations of the surface quantities.

Without bulk diffusion, linear stability with respect to spatially homogeneous perturbations is guaranteed if and only if the inequalities in (6.45) apply strictly. Within this context, a state may be stable in the presence of bulk diffusion and unstable otherwise. In this sense, we deduce that bulk diffusion has a stabilizing effect. The boundary between the collection of linearly stable and linearly unstable homogeneous states with respect to spatially homogeneous perturbations of the surface quantities is determined by a curve in the upper half of the $f_{u}g_{v}-f_{v}g_{u}$ versus $f_{u}+g_{v}$ plane. A plot, in figure 1, for representative choices of the bulk-surface coupling parameter β shows that the collection of stable homogeneous states increases with β.

#### (iii) Instability with respect to inhomogeneous perturbations

We now investigate the effect of inhomogeneous disturbances on a state $\left(U_{*},u_{*},v_{*}\right)$ that is linearly stable to homogeneous perturbations, as discussed earlier. In so doing, we recall that any such state corresponds to either of the cases delineated in (6.45) and (6.46).

For the steady state $\left(U_{*},u_{*},v_{*}\right)$ to be unstable to inhomogeneous disturbances, a growth rate ω>0 satisfying $G_{l}(\omega )=0$ must exist for some l>0. This being so, it can be shown that the condition that

6.47 $$f_{u}g_{v}-f_{v}g_{u}+d_{u}d_{v}{\left(l(l+1)\right)}^{2}+\beta l(d_{v}l(l+1)-g_{v})-l(l+1)(d_{u}g_{v}+d_{v}f_{u})<0$$

holds for some l>0 is sufficient to induce this kind of instability. When the state $\left(U_{*},u_{*},v_{*}\right)$ complies with case 1, $f_{u}+g_{v}\leq 0$ and, hence, the condition that (6.47) holds for some l>0 is not only sufficient but is also necessary for the existence of a growth rate ω>0 such that $G_{l}(\omega )=0$. This conclusion can be reached by investigating whether $G_{l}$ is a monotonic function of ω.

### (g) Numerical results

To confirm the stability analysis presented in the previous section, we used the COMSOL Multi-physics platform to conduct numerical simulations of the system (6.28) with f and g given by (6.30). In addition, we investigated the impact of the coupling parameter β on the response of the system. Our computational region was a unit sphere discretized by a mesh comprising 86 203 tetrahedral elements in the bulk and 21 120 triangular elements on the surface, with the unknown fields (U,u,v) approximated using piece-wise linear functions. To represent the perturbation of the homogeneous steady state $\left(U_{*},u_{*},v_{*}\right)$, we adopted the method of Krause *et al.* [24] and applied a random initial condition $\left(U_{0},u_{0},v_{0}\right)$ generated from a normal distribution with mean zero and standard deviation of $10^{-3}$. We then solved the system for various input parameters: α=1, λ=0, $d_{u}=1/400$ and $d_{v}=1/20$; β∈0,0.05,0.1,0.15; (ϵ,γϵ)∈(1.2,1.1),(0.9,0.81). For the selected parameter values, the system (6.28) has the unique homogeneous steady-state solution $\left(U_{*},u_{*},v_{*})=(0,0,0\right)$.

By (6.28), the surface reaction–diffusion equations are decoupled from bulk diffusion for β=0 and the conditions (6.45) and (6.47) are necessary and sufficient for surface diffusion-driven instability of the steady-state solution $\left(U_{*},u_{*},v_{*})=(0,0,0\right)$. This observation serves to distinguish the two sets of parameters adopted for ϵ and γ given that β=0, λ=0, $d_{u}=1/400$ and $d_{v}=1/20$: (i) (ϵ,γϵ)=(1.2,1.1): the steady-state solution $\left(U_{*},u_{*},v_{*})=(0,0,0\right)$ is stable in the absence of surface diffusion and unstable otherwise since both conditions (6.45) and (6.47) are satisfied; and (ii) (ϵ,γϵ)=(0.9,0.81): the steady-state solution $\left(U_{*},u_{*},v_{*})=(0,0,0\right)$ is unstable even in the absence of surface diffusion since condition (6.45) does not hold. This result is confirmed by our numerical simulations (not shown here), which in turn are in agreement with those presented by Krause *et al.* [24], from which the values $d_{u}=1/400$, $d_{v}=1/20$, ϵ=1.2 and γϵ=1.1 were taken.

**Figure 2.**
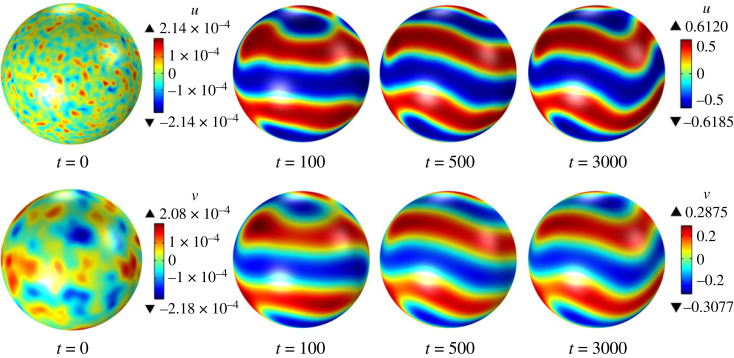
Time evolution of the surface quantities u and v obtained by solving the system (6.28) with ϵ=1.2, γϵ=1.1, λ=0, α=1, β=0.05, $d_{u}=1/400$ and $d_{v}=1/20$, starting from random initial conditions.

**Figure 3.**
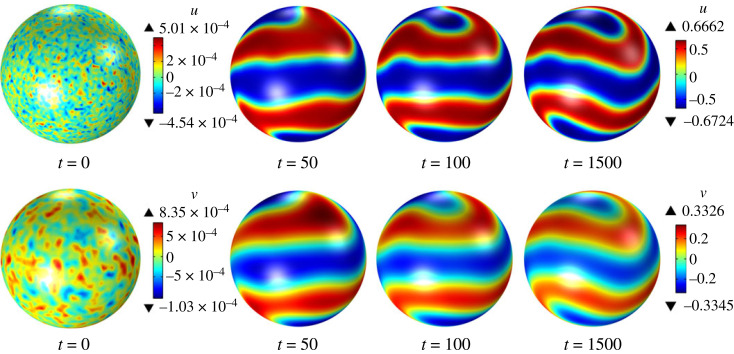
Time evolution of the surface quantities u and v obtained by solving the system (6.28) with ϵ=0.9, γ=0.9, λ=0, α=1, β=0.05, $d_{u}=1/400$ and $d_{v}=1/20$, starting from random initial conditions.

**Figure 4.**
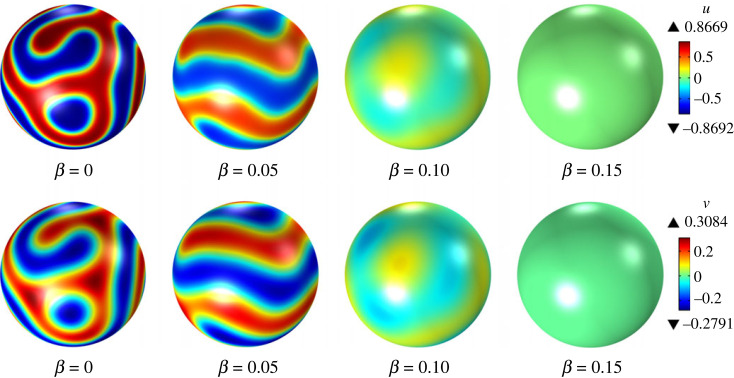
Steady pattern formation in response to spatially homogeneous perturbations for ϵ=1.2, γϵ=1.1, λ=0, α=1, $d_{u}=1/400$, $d_{v}=1/20$ and varying values of β.

**Figure 5.**
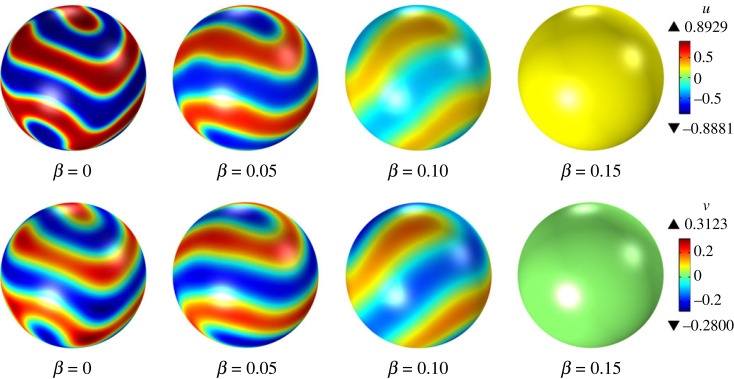
Pattern formation with respect to random initial conditions at a dimensionless time of t=500, for ϵ=0.9,γ=0.9, λ=0, α=1, $d_{u}=1/400$, $d_{v}=1/20$ and varying values of β.

**Figure 6.**
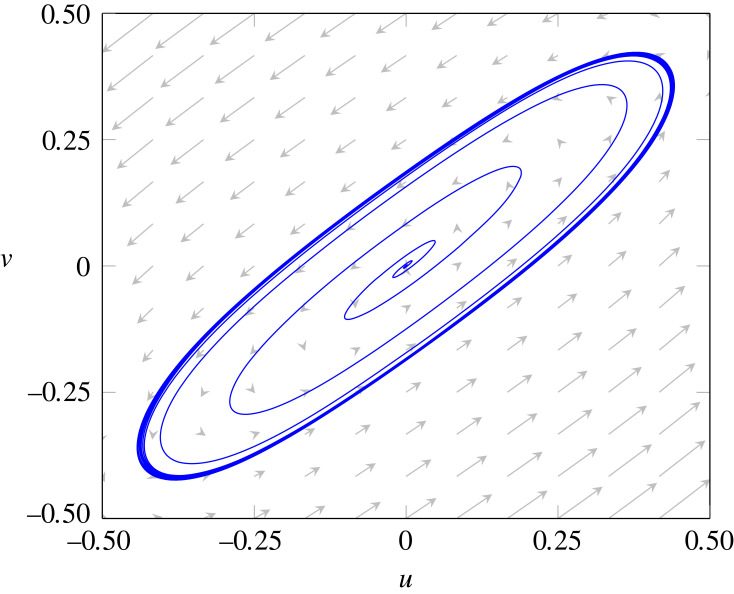
Phase portrait evaluated at the point (1,0,0) on the surface of the sphere for β=0.15 and fixed values of ϵ=0.9, γ=0.9, λ=0, α=1, $d_{u}=1/400$ and $d_{v}=1/20$.

For β>0, there are two sets of sufficient conditions for surface diffusion-driven instability, namely, (i) (6.45) and (6.47), and (ii) (6.46) and (6.47). For β=0.05, the first and second sets of sufficient conditions are satisfied for (ϵ,γϵ)=(1.2,1.1) and (ϵ,γϵ)=(0.9,0.81), respectively. Linear stability analysis predicts that surface diffusion-driven instability occurs in both cases. The time evolution of the surface fields u and v from initial random perturbations in our numerical simulations confirms this conclusion, indicating that the nonlinear reaction–diffusion system (6.28) exhibits pattern formation, as shown in figures 2 and 3. Our attention now shifts to the impact of increasing values of β on the long-term behaviour of the system (6.28). Figure 4 shows the steady-state distributions of u and v for (ϵ,γϵ)=(1.2,1.1), which are attained for all considered values of β. For β=0.0 and β=0.05, an initial random perturbation of the homogeneous state evolves into a steady-state that displays surface patterns resulting from the surface diffusion-driven instability mechanism of interest here. However, for β=0.10 and β=0.15, a random initial perturbation of the homogeneous state dies out over time. These observations support the notion that β acts to disrupt the spatial patterns due to the destabilizing effect of bulk diffusion. A similar behaviour is observed for (ϵ,γϵ)=(0.9,0.81), as depicted in figure 5. However, in this case, the steady state was not reached for any value of β, and therefore, we have shown the surface fields u and v at t=500. For β=0.15, the random initial state evolves into what appears to be a limit cycle characterized by sustained oscillations, as evidenced by the phase portrait, shown in figure 6, of the surface quantities u and v at the point with spherical coordinates (1,0,0). It is noteworthy that the spatial pattern observed for β=0 is not produced by the surface diffusion-driven instability mechanism since the homogeneous state is unstable in the absence of surface diffusion.

The system under consideration reached a steady state for all input parameters considered excepting β=0.15. To further illustrate the spatial patterns exhibited by the system in those steady states, we characterize those patterns using the structure factor associated with the surface field u. Specifically, we consider the structure factor S for the field $\varphi_{S}=u-\bar{u}$, where $\bar{u}$ is the average value of u on the spherical surface ∂R. This is defined by

6.48 $$S(k)=\frac{1}{4\pi }|\hat{\varphi }(k){|}^{2},$$

where k is the three-dimensional wave vector and where $\hat{\varphi }(k)$ is the Fourier transform of the field $\varphi (x)=\delta (r-1)\varphi_{S}(\theta ,\phi )$, with δ being the Dirac delta function and (r,θ,ϕ) being the spherical coordinates of x. For the sake of visualization, it is convenient to introduce the spherically averaged structure factor $S_{\text{avg}}$ through

**Figure 7.**
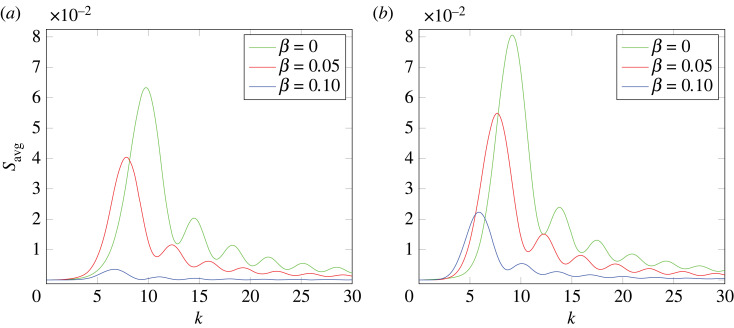
The spherically averaged structure factor $S_{\text{avg}}$ as a function of the magnitude k of the three-dimensional wave vector k for λ=0, α=1, $d_{u}=1/400$, $d_{v}=1/20$ and varying values of β for different values of ϵ and γ. (a) ϵ=1.2 and γϵ=1.1, and (b) ϵ=0.9 and γϵ=0.81.

6.49 $$S_{\mathrm{avg}}(k)=\frac{1}{4\pi }\int_{0}^{2\pi }\int_{0}^{\pi }S(k)\sin\tilde{\phi }\;\text{d}\tilde{\phi }\;\text{d}\tilde{\theta },$$

where $\left(k,\tilde{\theta },\tilde{\phi }\right)$ are the spherical coordinates of k. In figure 7, we present the spherically averaged structure factor, $S_{\mathrm{avg}}$, calculated under steady-state conditions. As the value of β increases, the peak values of $S_{\mathrm{avg}}$ decrease. When the patterns are fully suppressed, $S_{\mathrm{avg}}$ vanishes for all k. It is worth noting that the positions of the peaks are often used to characterize spatial patterns by determining their characteristic length scales. We also see that these peaks shift toward larger values of k as β decreases for both combinations of ϵ and γϵ considered.

## 7. Mechanochemical interplay

There are several scenarios where a single mobile constituent diffuses through the interstices of a deformable solid, with variations in constituent content leading to distortion of the solid or vice versa (see, for instance, Duda *et al.* [25,26], and the references therein). In this section, we will briefly discuss how to incorporate this particular kind of interplay into the previously developed framework. To accomplish this, we assume that the region R is occupied by a deformable solid body in its reference configuration, with constituent B able to move freely through the interstices of the solid while simultaneously inducing deformation. For simplicity, we limit our analysis to situations involving infinitesimal strains and rotations. Moreover, we assume that the constituents that comprise the solid through which constituent B diffuses are conserved and, thus, do not require explicit consideration in our analysis.

To account for the mechanical phenomenon of deformation, we first incorporate mechanics in the basic principles of theory. Accordingly, we must include the mechanical force balance in the list of the basic balances considered in our framework. The local version of that balance reads

7.1 $$\mathrm{div}\;T+b=0\;\text{in}\;R\;\text{and}\;Tn=s_{\mathrm{env}}\;\text{on}\;\partial R,$$

where T is the stress tensor, b is the external body force density and $s_{\text{env}}$ is the traction exerted upon R by the environment. It is concomitantly necessary to modify the bulk free-energy imbalance to include the power expenditures encompassing the actions of T, b and $s_{\text{env}}$. This culminates in an additional contribution to the bulk local version of the free-energy inequality (3.6) given by the negative of the stress power $T\cdot \dot{E}$, where

7.2 $$E=\frac{1}{2}(\nabla u+{\left(\nabla u\right)}^{\;\;⊤\;\;})$$

is the infinitesimal strain tensor and u is the displacement vector field.

To account for the solid distortion induced by variations in the content of constituent B, we take advantage of the additive decomposition

7.3 $$E=E_{e}+E_{c}$$

of the strain E into elastic $E_{\text{e}}$ and chemical $E_{\text{c}}$ parts, with

7.4 $$E_{c}=\frac{\upsilon }{3}(n_{B}-n_{\mathrm{ref}})I,$$

where υ, $n_{B}$ and $n_{\text{ref}}$ denote the molar volume, number density and referential number density of constituent B, respectively. With these considerations, it can be concluded that T and $\mu_{B}$ are given by

7.5 $$T=\frac{\partial \tilde{\psi }(E_{e},n_{B})}{\partial E_{e}}\;\text{and}\;\mu_{B}=\frac{\partial \tilde{\psi }(E_{e},n_{B})}{\partial n_{B}}-\upsilon \sigma ,$$

where σ=trT/3 is the mean stress. Under the assumption that the free-energy response is the sum of two parts, one elastic and the other chemical, with the elastic part appropriate to a linearly elastic and isotropic material and the chemical part given by (6.4)${\;}_{1}$, (7.5) specializes to

7.6 $$T=\lambda \;\text{ tr}\;E_{e}I+2GE_{e}\;\text{and}\;\mu_{B}=\mu_{B}^{0}+k_{B}T\ln\frac{n_{B}}{{\bar{n}}_{B}}-\upsilon \sigma ,$$

where λ and G are the Lamé moduli. By (7.3), (7.4) and (7.6)${\;}_{1}$, the mean stress σ can be expressed in terms of trE and $n_{B}$ as follows:

7.7 $$\sigma =\kappa (\text{ tr}\;E-\upsilon (n_{B}-n_{\mathrm{ref}})),$$

where κ:=(3λ+2G)/3 is the bulk elastic modulus.

To better understand the implications of the stress dependence of the chemical potential $\mu_{B}$ of constituent B, we can focus on the example discussed in the previous section. Firstly, we augment the expression of ${ȷ}_{B}$ given by (6.10)${\;}_{1}$ by a term proportional to the gradient ∇σ of the mean stress σ, thereby allowing for bulk diffusion to be driven also by stress. Furthermore, consistent with (6.13)${\;}_{1}$, ∇σ also affects the production of the adsorbed form of constituent B. In addition, since $\mu_{B}$ plays a central role in both fast and Marcelin–De Donder kinetics, its dependence on σ influences sorption processes. In particular, the extension of the dimensionless system (6.18) to account for the mechanochemical interplay reads

7.8 $$\begin{array}{l} \begin{array}{l} (\lambda +G)\nabla \text{ div}\;u+G△u-\kappa \upsilon {\bar{n}}_{B}\nabla b=0,\\ \dot{b}=D_{B}(△b-\frac{\upsilon }{k_{B}T}(b△\sigma +\nabla b\cdot \nabla \sigma )), \end{array}\}\;\text{in}\;R;\\ \begin{array}{l} (\lambda (\text{ div}\;u)I+G(\nabla u+{\left(\nabla u\right)}^{\;\;⊤\;\;})-\kappa \upsilon {\bar{n}}_{B}(b-b_{\mathrm{ref}})I)n=s_{\mathrm{env}},\\ \frac{\hat{K}(\sigma )b}{1+\hat{K}(\sigma )b}=a,\\ \dot{a}=D_{A}^{S}{△}_{S}a-\frac{D_{B}{\bar{n}}_{B}}{{\bar{n}}_{A}}(\nabla b-\frac{\upsilon b}{k_{B}T}\nabla \sigma )\cdot n+F(a,c),\\ \dot{c}=D_{C}^{S}{△}_{S}c+G(a,c), \end{array}\}\;\text{on}\;\partial R, \end{array}\}$$

where $\hat{K}$ is defined according to

7.9 $$\hat{K}(\sigma )=K\exp(-\frac{\upsilon \sigma }{k_{B}T}),$$

with K as introduced in (6.11) and $b_{\mathrm{ref}}:=n_{\mathrm{ref}}/{\bar{n}}_{B}$. The first condition in the system (7.8) is obtained by combining the mechanical force balance given by (7.1)${\;}_{1}$ in the absence of external body forces, the strain–displacement relation described by (7.2), the additive decomposition of strain expressed in (7.3), the expression for the chemical strain given by (7.4) and the constitutive relation for the stress tensor T as described by (7.6)${\;}_{1}$.

In view of (7.7), it is important to note that σ is determined by u and b. Hence, (7.8) provides a system of equations to be solved for the unknowns u, a and b. However, we choose to express the system so that the mean stress σ appears explicitly and to thereby highlight its role in the chemical processes in which constituent B participates.

Our decision to outline the mechanochemical interplay in the context of small deformations was deliberate. This was done not only to save space but also because infinitesimal strains and rotations are relevant and simple enough to reveal the relationship between mechanics and chemistry. By contrast, any effort to deal with finite strains and rotations would inevitably be accompanied by inherent complexities that could unduly obscure our understanding of the mechanochemical interplay. Nonetheless, we plan to address this issue in detail in a separate contribution. In this regard, the work of McBride *et al.* [27] is a valuable resource, as those workers have developed a general continuum framework that encompasses finite strains and rotations, bulk diffusion and surface diffusion.

## 8. Conclusion

We presented a framework for describing the interplay between the diffusion of a single chemical constituent within a bulk region and the chemical reactions and the diffusion of several chemical constituents on the boundary of that region. The coupling between the bulk and its boundary is established by the adsorption and desorption of the bulk constituent on the boundary, the adsorbed form of which participates in chemical reactions and diffusion. Our systematic derivation of the equations is based on a continuum framework in which basic balances are combined with constitutive relations that are consistent with a mechanical version of thermodynamics.

The derivation began by identifying the constituents that participate in the elementary reactions comprising all salient sorption and chemical reaction mechanisms. This was followed by the postulation of basic content balances that account for changes of composition due to diffusive transport, external supplies and production due to chemical reactions. The production rate of a given constituent was related to the kinetics of the chemical reaction it participates in, proportional to stoichiometric coefficients. It was found that the reaction rate can be described with recourse to two alternative kinetic schemes: fast or Marcelin–De Donder. For the former alternative, the reaction rates can be identified as Lagrange multipliers. For the latter alternative, the reaction rates are expressed in terms of the compositions of the salient constituents. Classical mass-action kinetics arises as a special case of general Marcelin–De Donder kinetics. Our treatment also encompasses situations in which various chemical constituents are subject to lattice constraints.

As an application, we derived the governing equations for bulk diffusion coupled with surface diffusion and reaction, with the kinetics of the FitzHugh–Nagumo type, assuming that sorption/desorption is very fast. We conducted a linear stability analysis around a homogeneous steady state and discussed sufficient conditions for surface diffusion-induced instabilities. Our results were corroborated through numerical simulations.

Our study focused on developing a general framework for deriving equations that describe the interplay between bulk diffusion and surface reaction and diffusion. We then performed a stability analysis and conducted simulations on a specific bulk-surface reaction–diffusion system arising from our framework. These studies were done with the tacit understanding that the system considered is solvable, at least for certain combinations of the material parameters that enter the various governing equations. The question of existence for bulk-surface reaction–diffusion systems has received significant attention in recent years. Relevant investigations include the works of Sharma & Morgan [28], Bothe *et al.* [29], Fellner *et al.* [30], Hausberg & Röger [31] and Morgan & Tang [32]. While we did not address the issue of the existence of solutions to the equations considered in our study, we acknowledge its importance and plan to address the issue in our future research.

Our framework has the potential to address various problems, ranging from solid-state hydrogen storage systems to cell biology. More importantly, it can be extended to incorporate multi-physics aspects such as mechanics, allowing us to deal with the complex interplay between mechanics and chemistry, which plays a crucial role in several scientific and technological phenomena. We illustrate such an extension for situations in which the bulk region is occupied by a deformable solid through which a single mobile constituent can diffuse.

## Data Availability

The data are provided in the electronic supplementary material [33].

## References

[1] Martin M, Gommel C, Borkhart C, Fromm E. 1996 Absorption and desorption kinetics of hydrogen storage alloys. J. Alloys Compd. **238**, 193-201. (10.1016/0925-8388(96)02217-7)

[2] Burkart T, Wigbers MC, Würthner L, Frey E. 2022 Control of protein-based pattern formation via guiding cues. Nat. Rev. Phys. **4**, 511-527. (10.1038/s42254-022-00461-3)

[3] Gomez D, Iyaniwura S, Paquin-Lefebvre F, Ward MJ. 2021 Pattern forming systems coupling linear bulk diffusion to dynamically active membranes or cells. Phil. Trans. R. Soc. A **379**, 20200276. (10.1098/rsta.2020.0276) 34743601

[4] Malevanets A, Kapral R. 1997 Microscopic model for FitzHugh–Nagumo dynamics. Phys. Rev. E **55**, 5657-5670. (10.1103/PhysRevE.55.5657)

[5] Fried E, Gurtin ME. 2007 Thermomechanics of the interface between a body and its environment. Cont. Mech. Thermodyn. **19**, 253-271. (10.1007/s00161-007-0053-x)

[6] Svehla G. 1993 Nomenclature of kinetic methods of analysis (IUPAC Recommendations 1993). Pure Appl. Chem. **65**, 2291-2298. (10.1351/pac199365102291)

[7] Feinberg M. 2019 Foundations of chemical reaction network theory. Cham, Switzerland: Springer.

[8] Houston PL. 2001 Chemical kinetics and reaction dynamics. New York, NY: McGraw-Hill.

[9] Gurtin ME, Fried E, Anand L. 2010 The mechanics and thermodynamics of continua. New York, NY: Cambridge University Press.

[10] de Gennes PG. 1980 Dynamics of fluctuations and spinodal decomposition in polymer blends. J. Chem. Phys. **72**, 4756-4763. (10.1063/1.439809)

[11] E W, Palffy-Muhoray P. 1997 Phase separation in incompressible systems. Phys. Rev. E **55**, R3844-R3846. (10.1103/PhysRevE.55.R3844)

[12] Coleman BD, Noll W. 1963 The thermodynamics of elastic materials with heat conduction and viscosity. Arch. Ratio. Mech. Anal. **13**, 167-178. (10.1007/BF01262690)

[13] Marcelin MR. 1915 Contribution à l’étude de la cinétique physico-chimique. Ann. Phys. **9**, 120-231. (10.1051/anphys/191509030120)

[14] De Donder T. 1922 L’Affinité. Applications aux gaz parfaits. Bull. Cl. sci., Acad. r. Belg. (5e sér.) **8**, 197-205.

[15] Van Rysselberghe P. 1958 Reaction rates and affinities. J. Chem. Phys. **29**, 640-642. (10.1063/1.1744552)

[16] Feinberg M. 1972 On chemical kinetics of a certain class. Arch. Ratio. Mech. Anal. **46**, 1-41. (10.1007/BF00251866)

[17] Gorban AN. 2013 Thermodynamics in the limit of irreversible reactions. Physica A **392**,1318-1335. (10.1016/j.physa.2012.10.009)

[18] FitzHugh R. 1961 Impulses and physiological states in theoretical models of nerve membrane. Biophys. J. **1**, 445-466. (10.1016/S0006-3495(61)86902-6) 19431309 PMC1366333

[19] Nagumo J, Arimoto S, Yoshizawa S. 1962 An active pulse transmission line simulating nerve axon. Proc. IRE **50**, 2061-2070. (10.1109/JRPROC.1962.288235)

[20] Turing AM. 1952 The chemical basis of morphogenesis. Phil. Trans. R. Soc. B **237**, 37-72. (10.1098/rstb.1952.0012)

[21] Murray JD. 2001 Mathematical biology II: spatial models and biomedical applications, vol. 3. New York, NY: Springer.

[22] Rätz A, Röger M. 2014 Symmetry breaking in a bulk-surface reaction-diffusion model for signalling networks. Nonlinearity **27**, 1805-1827. (10.1088/0951-7715/27/8/1805)

[23] Abramowitz M, Stegun IA (eds). 1972 Handbook of mathematical functions: with formulas, graphs, and mathematical tables. New York, NY: Dover Books on Mathematics.

[24] Krause AL, Burton AM, Fadai NT, Van Gorder RA. 2018 Emergent structures in reaction-advection-diffusion systems on a sphere. Phys. Rev. E **97**, 042215. (10.1103/PhysRevE.97.042215) 29758621

[25] Duda FP, Ciarbonetti A, Toro S, Huespe A. 2021 A phase-field model for solute-assisted brittle fracture in elastic-plastic solids. Int. J. Plast. **102**, 16-40. (10.1016/j.ijplas.2017.11.004)

[26] Duda FP, Souza AC, Fried E. 2021 Fluid flow and interface motion in gels: a finite-strain theory and its application to a channel flow problem. J. Mech. Phys. Solids. **155**, 104566. (10.1016/j.jmps.2021.104566)

[27] McBride AT, Javili A, Steinmann P, Bargmann S. 2011 Geometrically nonlinear continuum thermomechanics with surface energies coupled to diffusion. J. Mech. Phys. Solids. **59**, 21162133. (10.1016/j.jmps.2011.06.002)

[28] Sharma V, Morgan J. 2016 Global existence of solutions to reaction-diffusion systems with mass transport type boundary conditions. SIAM J. Math. Anal. **48**, 4202-4240. (10.1137/15M1015145)

[29] Bothe D, Köhne A, Maier S, Saal J. 2017 Global strong solutions for a class of heterogeneous catalysis models. J. Math. Anal. Appl. **445**, 677-709. (10.1016/j.jmaa.2016.08.016)

[30] Fellner K, Latos E, Tang BQ. 2018 Well-posedness and exponential equilibration of a volume-surface reaction-diffusion system with nonlinear boundary coupling. Ann. Inst. Henri Poincaré (C) Anal. Non Lineaire **35**, 643-673. (10.1016/j.anihpc.2017.07.002)

[31] Hausberg S, Röger M. 2018 Well-posedness and fast-diffusion limit for a bulk-surface reaction-diffusion system. NoDEA **25**, 1-32. (10.1007/s00030-018-0508-8)

[32] Morgan J, Tang BQ. 2022 Global well-posedness for volume-surface reaction-diffusion systems. Commun. Contemp. Math. **24**, 2250002. (10.1142/S021919972250002X)

[33] Duda FP, Forte Neto FS, Fried E. 2023 Modelling of surface reactions and diffusion mediated by bulk diffusion. Figshare. (10.6084/m9.figshare.c.6837198) PMC10645080 37926211

